# Tomes' Dental Surgery

**Published:** 1860-01

**Authors:** 


*Tomes' Dental Surgery.*—The American edition of this work greatly exceeds the English one in all the externals which have so much to do with one's comfort in reading. The cuts, we believe, are the same as those used in the English edition.

